# “They teach us how to look at the world”: student perceptions of traditional behavioral and underrepresented social-structural theoretical content in a graduate public health course

**DOI:** 10.3389/fpubh.2026.1893127

**Published:** 2026-10-19

**Authors:** Michael Harvey, Arden Castle, Vasundhara Mehta

**Affiliations:** 1 Department of Health Services, Policy and Practice, School of Public Health, Brown University, Providence, RI, United States; 2 Department of Public Health, San José State University, San José, CA, United States; 3 La Clinica de la Raza, Oakland, CA, United States

**Keywords:** behavioral theory, curriculum, education, social determinants of health, social theory, theory

## Abstract

**Introduction:**

Research and available teaching resources suggest that health behavior theories that seek to explain the health-relevant behaviors of individuals are commonly incorporated into US-based Master of Public Health (MPH) coursework, while social theories that seek to explain health-relevant social dynamics are much less prominent. Researchers have expressed concern that a disproportionate focus on individual behavior risks eliding social-structural factors in the patterning of health and disease.

**Methods:**

To address this gap in the public health curriculum, we piloted a public health theory course among MPH students that included both traditionally taught behavioral theories as well as underrepresented social and structural theories. To understand student perceptions of course content, we assessed their perspectives on the course and its content, as well as on their perceived relevance of course content to: (1) their future professional work and (2) addressing health inequalities. The assessment tool included Likert scales and open-ended questions. Analysis of answers to open-ended questions was informed by thematic analysis. The response rate was 100% (*n* = 17).

**Results:**

Students reported uniformly positive assessments of the course: all rated theoretical content as ‘good’ or ‘very good,’ and all considered the inclusion of social and structural theories ‘important’ or ‘very important.’ Similar levels of importance were attributed to behavioral theories. Students also indicated high perceived relevance of theory to future professional work and to addressing health inequities.

**Discussion:**

These findings provide actionable insights into student perceptions of theoretical course content. However, due to the limitations of this work (e.g., single assessment among a small group with no pre-course baseline or comparison group), they should be interpreted with some caution.

## Introduction

Theory plays a central, if sometimes unacknowledged, role in public health. Within the public health literature, theory is defined as “a set of interrelated concepts, definitions, and propositions that present a *systematic* view of events or situations by specifying relations among variables, in order to *explain* and *predict* the events or situations”‘(emphasis in original) (1), p. 26; (2), p. 9). Theory helps “explain causal connections between specified phenomena within and across specified domains by using interrelated sets of ideas” (39). In short, “[t]heory is explanation” (3) and it aids in our making sense of ourselves and the world around us.

Within the field of public health, theory is used to explain health-relevant phenomena and, by extension, to inform how these phenomena might be acted on and altered through public health interventions. For example, a theory might seek to explain a health-related behavior (e.g., cigarette smoking cessation) and predict how that behavior might be adopted, maintained, modified, or halted through public health interventions. Alternatively, a theory could seek to explain health-relevant social phenomena, structures, and processes, such as economic inequality, stigma, or racism. Such theories could also be used to inform efforts to address or mitigate these phenomena and thereby improve population health.

Research suggests that there exists a behavioral theoretical status quo within public health instruction. Goodson’s (4) review of the theoretical content of public health textbooks demonstrates substantial overlap in their behavioral theoretical content. This is echoed by Harvey and McGladrey’s (5) review of social and behavioral science MPH syllabi that showed that 93% of commonly taught theories in their sample were behavioral. Within textbooks commonly used in these courses, Westbrook and Harvey (6) further show a prevailing—although not exclusive—behavioral emphasis in how these classroom resources frame the relationship among health, behavior, and society. This accords with longstanding criticism of “psychological fundamentalism” (7) and behavioral “fantasy paradigms” (8, 9) within the field.

Despite this strong focus on health behaviors and behavioral theory within the classroom, social theory and social theoretical explanations for health have long played important, although less prominent, roles within public health. Social theories like fundamental cause theory (10), theory of gender and power (11), ecosocial theory and embodiment (12), social determination and subsumption (13, 14), political economy of health (15) and theories of class (16), structural violence (17) and structural vulnerability (18), critical race theory (19), intersectionality (20), structural racism (21), stigma (22, 23), and syndemics (24) seek to explain the social world—including social hierarchies, power, social structure, and discrimination—and their role in shaping health, disease, and their inequitable distributions over time and across diverse geographies. In describing these theories as ‘less prominent’ or underrepresented, we refer to their limited presence in MPH coursework relative to behavioral theory rather than their novelty. Indeed, systematic explanations for how society and social environments shape patterns of health and disease go back centuries and even millennia.

The social-ecological model, which describes the interacting levels that influence health outcomes (from the individual/intrapersonal level, to the institutional, community, and broader social-structural and policy environments) is widely incorporated in public health instruction, research, and practice (25). While behavioral theory is often appropriate for understanding and engaging with ‘lower levels’ of the ecological model, social theory is necessary for engaging with those ‘outer levels’ that concern community, social, and political dynamics. Moreover, overemphasizing behavioral theories of health (and, by extension, underemphasizing or neglecting social theories of health) can result in a narrowly behavioral understanding of why some are sick and others are healthy, thereby perpetuating the social drivers of health through their systematic analytic elision (26, 27). In response, some have called for greater incorporation of social theories into public health instruction, research, and practice (cf. (28, 29)).

With this backdrop in mind and the identified need for social theoretical instruction in public health education, we piloted a modified graduate-level public health theory course for MPH students enrolled in a program with a specialization in community health education. The course incorporated both traditional behavioral theories of health as well as underrepresented social and structural theories of health. Students were assessed on their general perspectives on the course and its theoretical content. Additionally, students were assessed on their perceived relevance of theoretical course content (1) to their future professional work and (2) to addressing health inequalities. Given the focus on better understanding student learning and experience, we situate this work within scholarship of teaching and learning (SoTL) (30).

This was a required (i.e., non-elective) course for students enrolled in this MPH program. As a result, course enrollment was not driven by students’ prior interest in theory or in health equity specifically. Enrolled students had both full- and part-time status, and they spanned various years in the program.

## Methods

### Theory identification

Course content was split between ‘traditional’ health behavior theory and ‘underrepresented’ social and structural theories of health. We defined health behavior theories as ones that focused principally on explaining health-relevant behaviors and on informing health behavior change. These theories are often concerned with individual-level factors—such as an individual’s beliefs, knowledge, motivation, and self-confidence—but not exclusively. On the other hand, social theories of health seek to explain society and health-relevant social phenomena, social structures, and processes.

Behavioral theoretical content was principally derived from the fifth edition of the widely used Glanz, Rimer, and Viswanath textbook, Health Behavior: Theory, Research, and Practice (31), which was assigned in the course. Other sources, such as the alternative health behavior theory textbooks mentioned above and work by Goodson (4) and Harvey and McGladrey (5), were also consulted to ensure the comprehensiveness of the behavioral theories incorporated in the course. Because there is no standard social-structural theoretical canon within public health instruction, the identification of these theories was more subjective and relied on the first author’s experience researching these topics and developing social theoretical public health courses.

In the process of identifying relevant course content, we adopted a broad view of theory that encompassed formal theories, theoretical concepts, models, and frameworks (terms that are admittedly often used interchangeably). For example, terms like self-efficacy (32) and structural violence (17), although they have been referred to as theories, might also be described as theoretical concepts. And popular “theories” are often referred to as models, such as the health belief model and the transtheoretical model. Other topics, like community organizing and community-based participatory research, might be referred to as theoretically informed approaches or methods, rather than theory per se. We also recognize that these terms are contested within public health and other fields (33, 34), and we sought to avoid engaging with these larger debates. For a list of the behavioral and social theories incorporated into the course, see Table 1.

**Table 1:** Theoretical content of course.

Traditional behavioral theories and related concepts	Underrepresented social theories and related concepts
Health belief model Theory of reasoned action Theory of planned behavior Integrated behavioral model Self-efficacy Transtheoretical model Precaution adoption process model Social cognitive theory Social support theory Social network theory Transactional model of stress and coping Health literacy Interpersonal communication Social marketing Media advocacy	Social gradient Social determinants of health Social-ecological model Behavioral justice Fundamental cause theory Structural determinants of health Structural violence Structural vulnerability Structural interventions Political economy of health Corporate determinants of health Life course theory Theories of racism Interpersonal racism / racial discrimination Cultural racism Structural racism Intersectionality Stigma Community engagement Community organizing Community building Community-based participatory research (CBPR) Popular epidemiology

### Assessment tool and data analysis

This research sought to assess MPH students’ perspectives on the course and its theoretical content, as well as their perceived relevance of traditional behavioral theory and underrepresented social theory to (1) their future work in public health and (2) addressing health inequalities and achieving health equity. An assessment tool was developed and piloted among two students, whose feedback was used to revise and finalize the tool, which consists of Likert scales and short-answer questions. The questionnaire was administered in-person during the final class session of the course during the Spring 2019 semester. Prior to administration, course content was reviewed with students, and students were provided a list of theories, frameworks, and concepts that had been covered during the semester to mitigate recall bias. Prior to administration of the questionnaire, consent for participation was obtained orally, during which students were told that participation was completely optional, would have no bearing on their course grade, and that student names would not be collected as part of the research. All students consented to participate in the research. The course instructor left the room during a 15-min period when students had the option to complete the questionnaire. This research was conducted in accordance with comparable ethical standards to the 1964 Declaration of Helsinki and its later amendments, as governed by the Belmont Report, US federal regulations (45 CFR 46), and San José State University’s Policy for the Protection of Human Subjects (S08-7). It was approved by the Institutional Review Board at San José State University (Protocol Number: F19134).

Descriptive statistics were generated from the responses to Likert scales. Themes from short-answer responses were developed according to thematic analysis methods as described by Braun & Clarke (35). The three authors separately familiarized themselves with short-answer responses and independently developed initial codes. The authorship team then collectively compared, refined, and finalized these into a shared set of codes. These codes were then organized under broader themes and sub-themes, which went through a series of reviews before being finalized. In accordance with reflexive thematic analysis, coding was an interpretive and iterative process, during which differences among coders were reconciled through open discussion, and no intercoder reliability statistic was sought.

## Results

All students enrolled in the course completed the questionnaire (*n* = 17). The first survey question asked students for an overall assessment of the course’s content: Overall, how do you rate the theoretical content of this course (i.e., the theories, models, and concepts that you learned)? Eleven students (65%) indicated ‘very good’ and six students (35%) indicated ‘good.’ As a follow-up question, respondents were also asked to “discuss why [they] chose that answer.” Responses fell into four broad themes: General relevance, applicability, affect, and content.

The ‘general relevance’ theme refers to general relatability of course content. Respondents indicated relevance of course content to various domains, including work, public health, health equity, life, and other coursework within the MPH program. Responses from eight students (47%) fell under this theme. Given that students indicated their perceived relevance of theory to various domains—not only their future work but public health broadly, health equity, other MPH coursework, and life beyond the program—it suggests they understood the relevance of this content to extend well beyond the bounds of a single course. The ‘applicability’ theme encompassed codes that specifically indicated the usefulness of the course content to work in public health, public health interventions, and public health research. Responses from five students (29%) fell under this theme. This suggests that a sub-set of students saw in the theoretical content practical usefulness in various dimensions of their future professional work.

The third theme, ‘affect,’ refers to expressed feelings about course content. These ranged from positive to ambiguous and negative. Four respondents (24%) expressed positive affect, with the majority stating that they found aspects of the content “enjoyable.” Others stated that the course improved their confidence in course topics. However, four respondents (24%) expressed ambiguous or negative affect about course content, including the feeling that the course topic (i.e., theory) was daunting or intimidating, confusing, difficult to apply, and that too much time was spent on some (unspecified) content. Some students expressed both positive and ambiguous or negative affect. Within codes organized under the theme of affect, students expressed positive, negative, and ambivalent sentiment about the course. This is a useful counterweight to some of the more uniformly positive perspectives about the course derived from the Likert scale ratings.

Finally, the ‘content’ theme encompassed a variety of codes that emerged from general comments about the course content. Answers from 10 respondents (59%) fell within this theme. Within this theme, three sub-themes were identified. The first is ‘ecological balance,’ which we applied to codes that referred to the theoretical diversity of the course, the inclusion of theories applicable at different ecological ‘levels,’ and the balance the course struck between traditional behavioral theories and underrepresented social-structural theories. Answers from four respondents (24%) reflected this sub-theme. The second sub-theme is ‘knowledge,’ which encompassed those codes that referred to the general importance of course content and expressions of increased knowledge through exposure to course content. Answers from seven respondents (41%) reflected this sub-theme. The third sub-theme is ‘materials,’ which encompassed codes that referred to course materials, including expressions of approval of the textbook (31) and of a document provided by the course instructor that summarized course concepts and theories. Answers from two respondents (12%) reflected this sub-theme. Of these sub-themes, ‘ecological balance’ speaks most directly to the intent of the course. Students found value in theories that spanned the various levels of the social-ecological model, and they appreciated the theoretical diversity of the course. One student left their response to this question blank.

For question #2, students were asked: Which theories (or concepts, frameworks, models, etc.) that you learned about in this course do you think are the most relevant to your future work in public health (beginning with the most relevant)? Respondents were permitted to list up to five theories. We first analyzed those theories that respondents indicated at the top of their list (i.e., the top-ranked “most relevant” theories). We characterize seven of these top-ranked “most relevant” theories as behavioral (comprising 41% of the top-ranked “most relevant” theories listed) and 10 as social-structural (comprising 59% of the top-ranked “most relevant” theories listed), as reported in Table 2. Considering all theories listed across ranks (84 listings in total), 34 (40%) were behavioral and 50 (60%) were social-structural.

**Table 2:** Top-ranked “most relevant” theories to students’ future professional work (Q2) and to addressing health inequalities (Q3) (*n* = 17 per question).

Theory (top-ranked response)	Future professional work (Q2), *n* (%)	Addressing health inequalities (Q3), *n* (%)
Behavioral theories		
Health belief model	4 (24%)	0 (0%)
Social cognitive theory	2 (12%)	0 (0%)
Self-efficacy	1 (6%)	0 (0%)
Subtotal, behavioral	7 (41%)	0 (0%)
Social-structural theories		
Social-ecological model	4 (24%)	3 (18%)
Theories of racism (incl. Cultural racism)	3 (18%)	1 (6%)
Social determinants of health	2 (12%)	6 (35%)
Structural determinants of health (incl. Structural interventions/models)	1 (6%)	4 (24%)
Community organizing	0 (0%)	2 (12%)
Fundamental cause theory	0 (0%)	1 (6%)
Subtotal, social-structural	10 (59%)	17 (100%)
Total	17 (100%)	17 (100%)

Cell values are the number and percentage of the 17 respondents (per question) whose top-ranked “most relevant” theory was the one listed; percentages are therefore calculated out of 17. These figures differ from the totals reported in the text, which count all theories listed across ranks (respondents could list up to five theories each); 84 such listings for Question 2 and 78 for Question 3. Theories that were not included by any student as top-ranked “most relevant” for Question 2 or Question 3 do not appear in the table. Subtotals may not sum to 100% due to rounding.

For question #3, students were asked: Which theories (or concepts, frameworks, models, etc.) that you learned about in this course do you think are the most relevant to addressing health inequalities and achieving health equity (beginning with the most relevant)? Here again, respondents were permitted to list up to five theories. And again, we first analyzed those theories that respondents indicated at the top of their list (i.e., top-ranked “most relevant” theories). We characterize all of these top-ranked “most relevant” theories as social-structural, as reported in Table 2. Considering all theories listed across ranks (78 listings in total), just 3 (4%) were behavioral and 75 (96%) were social-structural.

When the totality of theories listed by students is considered, the contrast between student perceptions of the kinds of theories most relevant to future work in public health and the kinds of theories most relevant to addressing health inequalities and achieving health equity is notable. Those theories seen as most relevant to future work are split roughly evenly between behavioral (40%) and social-structural (60%) theories, concepts, and frameworks (*n* = 84), while those seen as most relevant to addressing health inequities were almost exclusively social-structural (96%; *n* = 78).

For question #4, students were asked: Are there any theories (or concepts, frameworks, models, etc.) that you think should be excluded from future versions of this course? Students were also prompted, “If you do not think any of the theories should be excluded, please leave this blank.” Thirteen respondents left the question blank. One respondent wrote “liked all of them.” One respondent listed life course theory. One respondent listed health literacy. And one respondent listed life course theory, intersectionality, and interpersonal communication. While this question instructed respondents to leave the field blank if they did not think any theory should be excluded, students might have left the field blank for other reasons. We therefore do not interpret blank responses as an endorsement of the course content.

When asked question #5a, ‘How important was the inclusion of the commonly taught individual and interpersonal behavioral theories, as well as theories of communication and health literacy within this course?’ students attributed high levels of import to these traditionally taught theories. Thirteen respondents (76%) indicated ‘very important’ and two respondents (12%) indicated ‘important.’ Two respondents (12%) did not provide a response. As a follow-up question, students were asked to “discuss why [they] chose that answer.” Codes were grouped into three broad themes, which included: (1) Important content; (2) Professional relevance; and (3) Sequencing. Responses from six students (35%) fell under the ‘important content’ theme. These codes expressed the general importance of this content and its specific importance to understanding public health and related topics, like individual choices and community needs. Responses from eight students (47%) fell under the ‘professional relevance’ theme. These codes referred to the relevance of this content specifically to future work in public health and to developing public health interventions. Responses from eight students (47%) fell under the ‘sequencing’ theme. These codes referred to the helpfulness of this content as an introduction to theory and a useful precursor to the “more complex” (according to some respondents) social-structural theories. Taken together, these responses suggest that students valued behavioral theory not only for its inherent importance to the field of public health and its usefulness to future work in the field, but also as a pedagogical stepping-stone to later course content related to social-structural theory.

When asked question #5b, ‘How important was the inclusion of social and structural theories within this course?’ students attributed high levels of import to these theories as well. Eleven respondents (65%) indicated ‘very important’ and four respondents (24%) indicated ‘important.’ Two students did not provide a response. As a follow-up question, students were asked to “discuss why [they] chose that answer.” Qualitative codes were grouped into broader themes, which included: (1) Professional relevance; (2) Social determinants of health; and (3) Alternative lens. Responses from eight students (47%) fell under the ‘professional relevance’ theme. These codes referred to the general relevance of this content and specifically to work in public health. Responses from nine students (53%) fell under the ‘social determinants of health’ theme. These codes broadly referred to the ways in which social and structural theory helped students to better understand the social determinants of health. Responses from four students (24%) fell under the ‘alternative lens’ theme. These responses discussed the ways in which social and structural theory provided new and more comprehensive ways of seeing the world. One student commented that this content “[a]llow[s] us to think on a deeper level about issues we see.” Another student commented that “[t]hey teach us how to look at the world.” A third student commented that they are helpful for thinking “upstream,” referring to the commonly used upstream-downstream metaphor, wherein upstream action is associated with preventive measures and addressing the root causes of disease. These themes are notable for their similarity to themes present in Question 5a, as well as their distinctiveness. Like their assessment of individual and interpersonal behavioral theories in Question 5a, students indicated high perceived relevance of social and structural theories to their work in public health. However, students also expressed a more unique aspect of social and structural theory—that they provide insights into the social determinants of health, a cornerstone of public health. Moreover, a subset of students understood social and structural theory as generative of new and important perspectives of the world.

## Discussion

These findings indicate roughly equal interest in, and perceived relevance of, both social-structural and behavioral theories among a group of MPH students enrolled in a program with a specialization in community health education. While more research among a larger group of students with more diverse topical interests is warranted, these findings suggest that social-structural theoretical instruction within US graduate public health training might be positively received by students. What was particularly notable in our results was the difference in student responses to Questions 2 and 3; while students gave roughly equal weight to the relevance of behavioral theory and social-structural theory when considering their future work in public health, they almost exclusively indicated social-structural theories when considering those most relevant to addressing health inequalities and achieving health equity. At a time when topics related to health equity have become central to public health education, our findings suggest that social theoretical instruction can play an important role in addressing these topics in the classroom. Also, by extension, educational programs that exclude social-structural theories might be perceived by students as inadequately preparing them to address issues of inequality.

This work has a number of limitations. As noted, these findings reflect the perspectives of only 17 students enrolled in a single course in an MPH program with a specialization in community health education. The research consists of a post-only questionnaire with no pre-course baseline or comparison group. It was administered in class on the final day of the course. While students’ positive response to course content could reflect the course content itself, it may also reflect desirability bias or measurement conditions. And given the focus of the program on community health education, students may have been predisposed to theoretical instruction generally, and social-structural theoretical instruction specifically. Question #4 asked students to leave the field blank if they did not think any theory should be excluded. However, because a blank response cannot be distinguished from a skipped or overlooked item, we cannot interpret a blank response as an endorsement of course content. Future iterations of the instrument should be modified to address this deficiency. Moreover, the assessment instrument was piloted among only two students before use; greater attention should be paid to study tool validation prior to future use.

In addition, in trying to accommodate both behavioral and social-structural theory, this course arguably tried to incorporate too much content, although such sentiments were not expressed by students in the data collected. In order to avoid overwhelm among students, curriculum committees, program directors, and instructors might consider distributing theoretical instruction across required coursework rather than concentrating it within just one or two courses. Relatedly, our assessment captured students’ exposure to this content and their general perceptions of it, not their degree of understanding of this content or ability to apply these theories in research or program planning. Future evaluations would benefit from measures of learning and application rather than only perceived relevance.

## Conclusion

Theory has been unevenly incorporated into public health instruction. In order to provide a more balanced introduction to public health theory, we piloted a course that incorporated both behavioral health theory and social-structural health theory. Our findings show that students express positive sentiment toward both behavioral and social-structural theory, and they express high levels of perceived relevance of both sets of theories to their future work in public health. Students almost exclusively listed social-structural theories when indicating those theories most relevant to addressing health inequality and achieving health equity.

By incorporating social-structural theory within course content, instructors can begin to address longstanding critiques of public health for its sometimes inappropriately narrow theorization of individuals, their behavior, and “lifestyle,” and its relative neglect of social and structural theory. Public health courses that only incorporate behavioral theory could inculcate among future public health practitioners an understanding of disease etiology, distribution, and prevention in largely behavioral terms. This not only risks eliding the social-structural drivers of disease (i.e., inequitable policies, systems, institutions, and harmful social, political, and economic forces), it also risks overinvestment in behavioral change that unduly burdens individuals with the responsibility for change (26, 27). Ultimately, the absence of social-structural theoretical instruction leaves public health professionals ill-equipped to explain or address the pathogenic forms of social organization, structures, and hierarchy that produce disease and inequitably distribute it, often along lines of class, race, gender, sexuality, ability, citizenship status, and nationality, among other social categories. Currently, there is no consensus regarding a theoretical “canon” for social theoretical instruction, and there is a pressing need for some consensus regarding the social theories that should be incorporated into graduate public health instruction.

The movement toward greater emphasis on social theory and structural determinants of health could be part of a broader embrace of structural competency within the field of public health (36). Defined as the trained ability “to recognize and respond to disease and its unequal distribution as the outcome of harmful social structures, such as policies, institutions, and systems” (36, 37), structural competency-based curricula are being actively developed for US-based medical education and other clinical professions (27, 38). The incorporation of social-structural theory into public health curricula could play a central role in developing structural competency among public health trainees.

## Data Availability

The original contributions presented in the study are included in the article/supplementary material, further inquiries can be directed to the corresponding author/s.
